# *GNAS* mutation detection in circulating cell-free DNA is a specific predictor for intraductal papillary mucinous neoplasms of the pancreas, especially for intestinal subtype

**DOI:** 10.1038/s41598-020-74868-2

**Published:** 2020-10-20

**Authors:** Tatsuo Hata, Masamichi Mizuma, Fuyuhiko Motoi, Yuko Omori, Masaharu Ishida, Kei Nakagawa, Hiroki Hayashi, Takanori Morikawa, Takashi Kamei, Toru Furukawa, Michiaki Unno

**Affiliations:** 1 Department of Surgery, Tohoku University Graduate School of Medicine, 1-1, Seiryo-machi, Aoba-ku, Sendai, Miyagi 980-8574 Japan; 2 Department of Investigative Pathology, Tohoku University Graduate School of Medicine, Sendai, Japan

**Keywords:** Pancreatic disease, Diagnostic markers, Tumour biomarkers, Pancreatic cancer

## Abstract

Pancreatic cystic neoplasms (PCNs) are a heterogeneous group with varying risks of malignancy. To explore the clinical utility of liquid biopsy in cyst type classification, we analyzed the *GNAS/KRAS* mutations in circulating cell-free DNA (cfDNA) obtained from 57 patients with histologically diagnosed PCNs, including 34 with intraductal papillary mucinous neoplasms (IPMNs) and compared the mutant allele prevalence and variant patterns with the paired resected specimens using next-generation sequencing. The positive prevalence of *GNAS* mutations in cfDNA of patients with IPMN (n = 11, 32%) was significantly higher than that in those with other PCNs (0%, *P* = 0.002). Conversely, *KRAS* mutations were detected in cfDNA of only 2 (6%) IPMN patients. The paired-sample comparison revealed highly concordance between the *GNAS* mutation status of cfDNA and resected IPMN specimens. Similar distributions of *GNAS* mutation positivity in cfDNA were observed across the different histological grades, whereas IPMNs with intestinal subtype showed a significantly higher prevalence of *GNAS* mutations than other subtypes (*P* = 0.030). *GNAS* mutation positivity in cfDNA was significantly associated with the acellular mucin pool of histological findings in primary IPMN lesions (*P* = 0.017). Detection of *GNAS* mutation in cfDNA can serve as a novel biomarker for cyst type classification and differentiation of intestinal subtype IPMN from the other PCNs.

## Introduction

The prevalence of pancreatic cystic neoplasms (PCNs) has been increased due to a consequence of the greater use of high-quality, cross-sectional abdominal imaging^1^. PCNs represent heterogeneous group of diseases with variable malignant potential and several clinical guidelines have been adopted to assist clinicians in determining the management in each cyst type^2–4^. Serous cystic neoplasms (SCNs) are usually benign and have low potential for malignancy, and when asymptomatic, can be managed through observation. However, resection at the time of diagnosis without any surveillance is recommended for mucinous cystic neoplasms (MCNs) and solid pseudopapillary neoplasms (SPNs), both of which have high malignancy potential. Intraductal papillary mucinous neoplasms (IPMNs) exhibit a wide spectrum of histological transformation ranging from low-grade dysplasia (LGD) to invasive carcinoma (INV). Only high-grade dysplasia (HGD) and INV are recommended to be resected and LGD should be managed by surveillance^5–7^. Taken together, the major challenges for the better practical management of PCNs is the accurate stratification of cyst type and their risk assessment for the presence of malignancy. Currently, a set of clinical and radiologic parameters based on the clinical guidelines are used to diagnose, risk-stratify, and manage PCNs^3,5,8^. Nevertheless, there is a need for better identification of cysts with malignant potential. Pancreatic resection is an invasive procedure and it is associated with a significant risk of morbidity and mortality, which may be unnecessary when the cyst has no malignant potential. Therefore, finding potential reliable predictors for cyst type classification and malignancy risk must be the focus for clinicians.

Previous reports have revealed potential biomarkers in predicting cyst type and malignancy risk^9–11^. The PCNs were well-characterized by the mutational profiles with high specificity in each cyst type^12^. For example, *VHL* alterations were unique to SCNs, *CTNNB1* mutations were found in SPNs and *GNAS* mutations in IPMNs^13^. The identification of these PCN-associated genes from patients’ samples, such as cyst fluid and pancreatic juice samples, provides strong diagnostic information for the classification of cyst type^14,15^. As a less-invasive and simpler approach, detection of gene mutations in circulating cell-free DNA (cfDNA) from the patients with PCNs was investigated by Berger and colleagues, and they demonstrated that the *GNAS* mutant alleles were detectable specifically in IPMN patients with high positive prevalence (~ 70%), but not *KRAS* mutants^16^. However, no data were available regarding the differences in *GNAS*/*KRAS* mutant positivity in the cfDNA of patients with different histological grades of IPMNs. Furthermore, the reasons for the markedly higher prevalence of *GNAS* mu tations than *KRAS* in the cfDNA remain unclear.

Herein, we aimed to clarify the utility of *GNAS*/*KRAS* genotyping using cfDNA to identify the IPMNs differentiating from other PCNs and to segregate high-risk lesions. Furthermore, to explore the pathogenesis of the *GNAS* mutant allele presence in their systemic circulation, we also evaluated the clinical and histological features of primary IPMN lesions in the cases representing the *GNAS* mutant alleles in cfDNA.

## Results

A total of 57 enrolled patients underwent pancreatic resection and all PCNs were histologically proven in terms of cyst type classification and grade of dysplasia. Of the 57 PCNs, 34 patients had IPMN, 9 had MCN, 10 had SPN, and 4 had SCNs (Table 1). Clinical features of the age, sex, and tumor location in each cyst type were similar to previous evidence^2^. The PCNs with HGD/INV were found in 27 of 34 IPMN patients and only one of 9 MCN patients (Table 1).

**Table 1:** Patient characteristics according to the cyst type.

Characteristics	IPMN (n = 34)	MCN (n = 9)	SPN (n = 10)	SCN (n = 4)
Male/Female (n)	20/14	0/9	1/9	1/3
Age, median (range), year	70 (46–87)	48 (35–59)	34.5 (22–64)	51 (42–62)
**Cyst location (n)**				
Head/body and tail	25/9	0/9	0/10	0/4
Cyst size, median (range), mm	35 (10–90)	48 (30–170)	33.5 (10–160)	26.5 (8–28)
**Mural nodule ≥ 5 mm (n)**				
Absent/Present	13/21	7/2	1/9	3/1
**Dilatation of MPD ≥ 10 mm (n)**				
Absent/Present	26/8	9/0	10/0	4/0
Dilatation of MPD ≥ 5 mm (n)				
Absent/Present	13/21	9/0	10/0	4/0
**Preoperative diagnosis**				
PDAC/IPMN/MCN/SPN/SCN	4/30/0/0/0	0/0/9/0/0	0/0/0/10/0	0/1/2/0/1
**Morphological duct type (n)**				
Main duct/mixed/branch duct	10/8/16			
**CT/MRI findings**				
High-risk stigmata	24			
Worrisome features	9			
No concerning features	1			
**Operative procedure (n)**				
PD/DP/TP/MP	23/8/2/1	0/9/0/0	0/8/0/2	0/3/0/1
**Histological grade (n)**				
LGD/HGD/INV	7/16/11	8/1/0		
**Histological subtype (n)**				
GAS/INT/PB/ONC	14/15/3/2			

PDAC, Pancreatic ductal adenocarcinoma; IPMN, Intraductal papillary mucinous neoplasms; MCN, Mucinous cystic neoplasm; SPN, Solid pseudopapillary neoplasm; SCN, Serous cystic neoplasm; MPD, Main pancreatic duct; PD, Pancreaticoduodenectomy; DP, Distal pancreatectomy; TP, Total pancreatectomy; CP, Central pancreatectomy; LGD, Low-grade dysplasia; HGD, High-grade dysplasia; INV, Invasive carcinoma; GAS, gastric subtype; INT, intestinal subtype; PB, pancreatobiliary subtype; ONC, oncocytic subtype.

**Figure 1:**
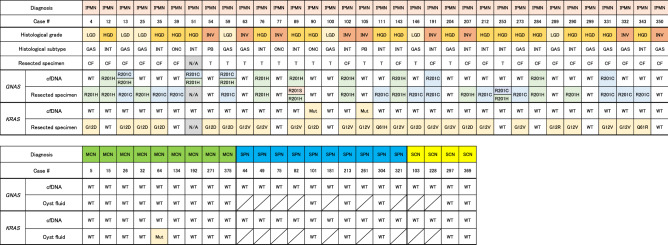
Illustration of overall mutational status in cell-free DNA and resected specimens for all enrolled patients with pancreatic cystic neoplasms. IPMN, intraductal papillary mucinous neoplasm; MCN, mucinous cystic neoplasm; SPN, solid-pseudopapillary neoplasm; SCN, serous cystic neoplasm; LGD, low-grade dysplasia; HGD, high-grade dysplasia; INV, invasive carcinoma; GAS, gastric subtype; INT, intestinal subtype; PB, pancreatobiliary subtype; ONC, oncocytic subtype; N/A, not applicable; CF, surgically aspirated cyst fluid sample; T, tussue sample. cfDNA, circulating cell-free DNA; WT, wild-type; Mut, mutant positive.

**Figure 2:**
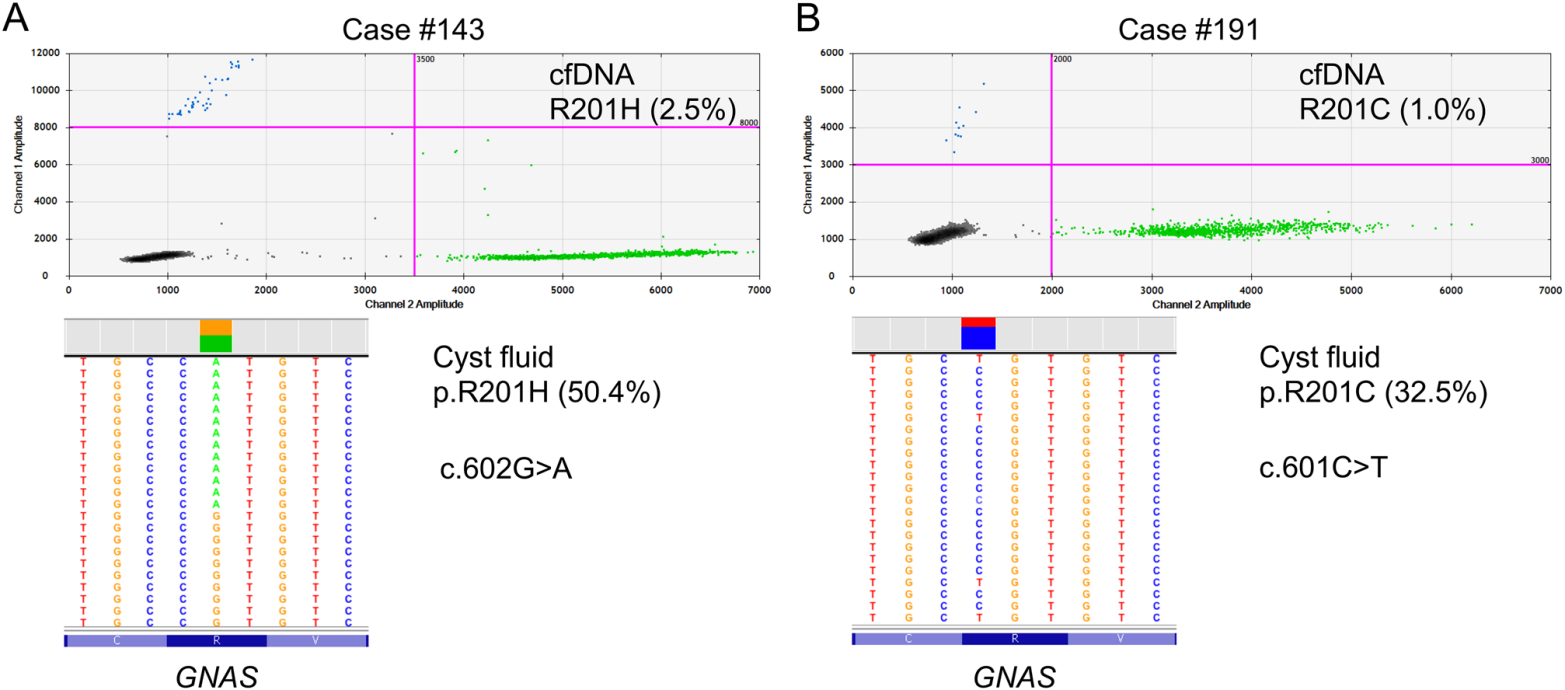
Representative cases harboring *GNAS* mutation of R201H (**A**) and R201C (**B**) in both cfDNA and resected specimen. In the upper side of 2-D plot, pink lines showed the thresholds of fluorescent value of FAM (blue droplets, mutant type) and HEX (green droplets, wild-type). Lower illustration in each case is a representation of the reads aligned to the reference genome, as provided by the Integrative Genomics Viewer (IGV) software for the hotspot mutations in the GNAS codon 201.

Figure 1 shows the overall *GNAS/KRAS* mutations detected in cfDNA and paired resected specimens of surgically aspirated cyst fluids or tissues from the patients with PCNs. Only one case (#51), with low-quality and unavailable tissue sample, was excluded from further genetic analysis. Next-generation sequencing revealed *GNAS* and *KRAS* mutations were found in 23 (70%) and 25 (76%) of the 33 IPMN resected specimens, respectively (Fig. 1). The median yields of cfDNA in plasma was 7.5 ng (range 3.0–24.4) per mL of plasma. The *GNAS* mutations in cfDNA were detected in 11 (32%) of the all 34 IPMN patients and 10 (43%) of the 23 IPMN patients harboring *GNAS* mutation in their primary lesions (Fig. 1). The representative cases harboring *GNAS* mutation in cfDNA are shown in Fig. 2. Paired comparison of mutant allele frequencies (MAFs) of *GNAS* between cfDNA and resected specimen revealed the much higher MAF in resected specimen than cfDNA; however, not all cfDNA positive cases showed the highly abundance of *GNAS* mutant alleles in their primary lesion (Supplementary Figure S1). No *GNAS* mutations were identified in cfDNA from the patients with non-IPMN PCNs of MCN, SPN, and SCN. The *KRAS* mutations in cfDNA were detected in only 2 (6%) of the 34 IPMN patients and both of two also had *KRAS* mutation in their IPMN lesions (Fig. 1). No *KRAS* mutations were detected in cfDNA of patients with non-IPMN PCNs. One case (#64) with MCN with LGD harbored the 7% of *KRAS* mutant alleles in the surgically aspirated cyst fluid.

We then compared the prevalence of *GNAS* mutant positivity in cfDNA with that in paired resected specimen. All IPMN cases positive for *GNAS* mutations in cfDNA harbored identical mutation patterns in their resected specimens. All IPMN cases without *GNAS* mutations in their resected specimens had only *GNAS* wild-type alleles in their cfDNA. Of the 11 cases with *GNAS* mutation positive in their cfDNA, heterogeneous mutations were detected in 3 cases. Among these 3 cases, 1 (#59) had apparent multicentric branch-duct IPMNs with dilatation of main pancreatic duct, suggesting the possibility of polyclonality across the multicentric IPMNs (Supplementary Figure S2).

**Figure 3:**
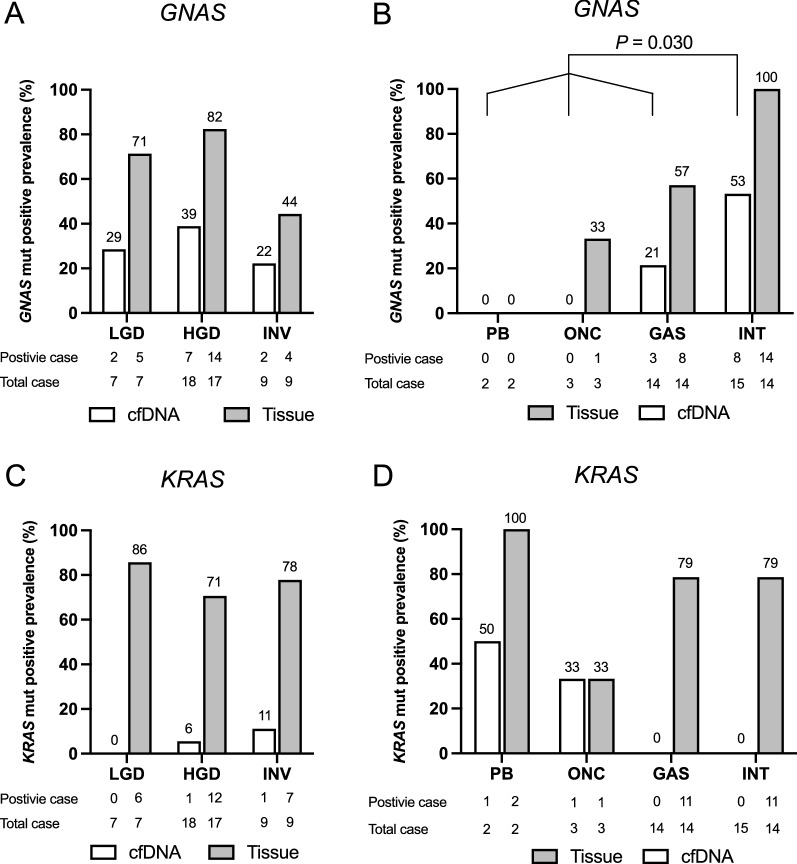
Positive prevalence of *GNAS* mutations in cell-free DNA and resected specimens according to the histological grade (**A**) and histological subtype (**B**). Likewise, *KRAS* mutant positivity in cfDNA stratified by the histological grade (**C**) and subtype (**D**). LGD, low-grade dysplasia; HGD, high-grade dysplasia; INV, invasive carcinoma; PB, pancreatobiliary subtype; ONC, oncocytic subtype; GAS, gastric subtype; INT, intestinal subtype. cfDNA, circulating cell-free DNA.

**Figure 4:**
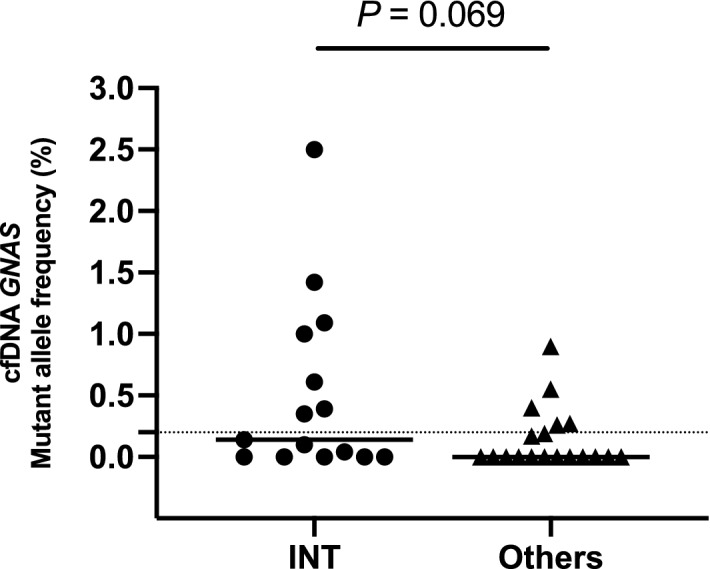
The *GNAS* mutant allele frequency in cfDNA from the IPMN cases with or without intestinal subtypes. The longer horizontal bar represents the median value. Dotted line represents the 0.2%, approximately correspondence to 2 positive droplets. cfDNA; circulating-ceil free DNA; INT, intestinal subtype. cfDNA, circulating cell-free DNA.

Despite the strong concordance of *GNAS* mutational patterns between cfDNA and tissue specimens of the same individual, not all IPMN cases harboring *GNAS* mutations in their primary lesion presented *GNAS* mutation positive in cfDNA. We then evaluated the specific features of IPMNs harboring *GNAS* mutations in cfDNA. No significant differences of clinical findings of primary IPMN lesions were seen between the cases with *GNAS* mutant positive in cfDNA and negative (Supplementary Table S1). The positive prevalence of *GNAS* mutation in cfDNA showed similar distribution across the different histological grades (Fig. 3A). With respect to histological phenotype of mucin-hypersecretion, *GNAS* mutation detection in cfDNA had a significantly higher positive prevalence in cases with intestinal subtype than in cases with other subtypes (*P* = 0.030, Fig. 3B). Although their prevalence was low (only two positive cases), *KRAS* mutant alleles were detected in cfDNA of only cases with HGV/INV (Fig. 3C). Moreover, the IPMN cases with intestinal subtype showed a trend of higher values of *GNAS* mutant allele frequencies in their cfDNA than those with other subtypes (*P* = 0.069, Fig. 4). In contrast to the *GNAS*, no IPMN cases with intestinal subtypes showed *KRAS* mutant alleles in cfDNA (Fig. 3D).

To test whether the *GNAS* mutant alleles detected in cfDNA were derived from the primary IPMN lesions, we compared the positive prevalence and allele frequency of the *GNAS* mutant between pre- and post-operative pairs of blood samples. Among the 10 evaluable cases, only 3 showed the *GNAS* mutation in cfDNA before pancreatic resection. After pancreatic resection, no *GNAS* mutations were found in any of the 3 cases (Table 2).

**Table 2:** *GNAS* mutant allele frequencies using 10 pairs of pre and postoperative blood samples.

Case #	Mutational pattern	Mutant allele frequency (%)	
		Pre-ope	Post-ope
4	R201C/R201H	0.00	0.00
63	R201C/R201H	0.00	0.00
89	R201H	**0.27**	0.00
143	R201H	**2.50**	0.00
146	R201C/R201H	0.00	0.00
191	R201C	**1.00**	0.00
204	R201C/R201H	0.00	0.00
207	R201C/R201H	0.00	0.00
212	R201C/R201H	0.00	0.00
253	R201C/R201H	0.00	0.00

**Figure 5:**
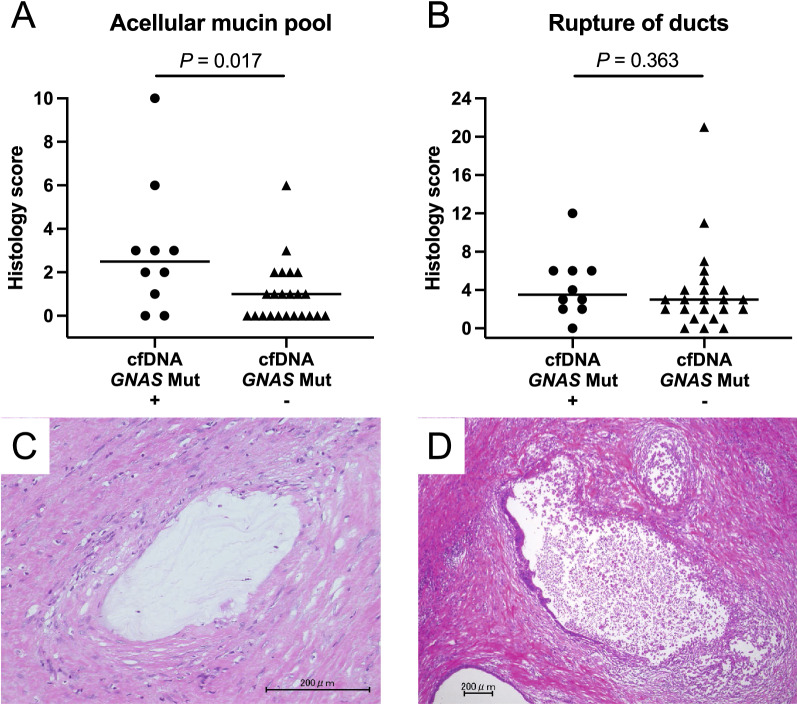
Association of *GNAS* mutant positivity in cfDNA and histological findings of primary IPMN lesions. The presence of acellular mucin pool (**A**) and rupture of ducts (**B**) in cases with or without *GNAS* mutation in their cfDNA. Representative histological images of acellular mucin pool (**C**) and rupture of ducts (**D**).

Based on the highly prevalent *GNAS* mutation in cases of IPMN with intestinal subtype, we evaluated the association between *GNAS* mutant positivity in cfDNA and local histological findings. The presence of acellular mucin pool lacking neoplastic epithelium was more frequently found in primary lesions from the IPMN cases with *GNAS* mutant positive cfDNA than those with negative (*P* = 0.017, Fig. 5A,C). Rupture of pancreatic ducts was also evaluated, and no significant difference in frequency was observed between the IPMN cases with *GNAS* mutant positive and negative in cfDNA (*P* = 0.363, Fig. 5B,D).

## Discussion

Appropriate management of patients with PCNs depends on the accurate classification of the cysts. Because of the increasing detection of asymptomatic PCNs, a novel non-invasive, repeatable, and reliable diagnostic test are urgently needed. Despite the insufficiently accurate for predicting the histological grades of IPMN, *GNAS* mutation detection in plasma cfDNA from patients with PCNs can be an evolving tool distinguishing IPMN from other types of PCNs such as MCN, SCN, and SPN. Furthermore, to the best of our knowledge, this is the first study to report following the two notable findings: first, mutant *GNAS* in cfDNA was derived from the primary IPMN lesion based on the genetic analysis using the resected specimens and pre- and post-operative blood samples. Second, *GNAS* mutant positivity in cfDNA is attributable to the histological disruption and mucin-hypersecretion at the primary IPMNs, especially known as intestinal subtype.

Despite the lower prevalence, the *GNAS* mutation pattern of missense substitution in cfDNA was highly concordant with the resected specimens. This result suggests that not all IPMNs harboring *GNAS* mutation are detectable by liquid-biopsy; however, *GNAS* mutation detection in cfDNA is a 100% positive predictive value for the presence of IPMN since there was no IPMN case in which *GNAS* mutation was positive in the cfDNA but negative in tissues. Furthermore, no *GNAS* mutations were detected from available postoperative blood samples. Taken together, *GNAS* mutant in cfDNA, definitively derived from the primary lesions harboring the identical mutation, can serve as a specific predictor of IPMN differentiating from other PCNs.

A few cases showed mutational heterogeneity of *GNAS* in cfDNA even though homogeneous mutation patterns were found in the primary IPMN lesions. As shown in Supplementary Figure S2, only one patient (#59) with multiple IPMNs underwent pancreatectomy for only high-risk lesions in body and tail of the pancreas. This patient harbored the low-risk branch-duct IPMN in the remnant pancreas head. Regrettably, a postoperative blood sample was unavailable and, therefore, it was difficult to further investigate whether the mutational heterogeneity of *GNAS* in cfDNA is mainly attributable to the difference in the mutation pattern between the resected and remained IPMNs. One more possible explanation for the heterogeneous mutations in cfDNA is presence of minute and invisible IPMN, the so-called incipient IPMN, in the remnant pancreas. Another possible reason is intratumoral heterogeneity with polyclonal nature during the unifocal IPMN development.

Our study is notable for the significant difference in the positive prevalence between the *KRAS* and *GNAS* mutations in cfDNA. Despite the similar positive prevalence between the *KRAS* and *GNAS* mutations (70–80%) in the resected IPMN specimens, a much lower prevalence of *KRAS* mutation than that of *GNAS* was detected in cfDNA. Recent studies have revealed that circulating pancreatic epithelial cells were found in peripheral blood samples collected from the patients of IPMN without invasion; however, the *GNAS*/*KRAS* mutational status for those circulating cells is still unelucidated^17–19^. Definitive reasons remain unclear; however, quite different prevalence of mutant positivity between *KRAS* and *GNAS* in cfDNA can be explained by a difference in biological signatures according to the histological subtypes.

Intestinal subtype IPMN, highly prevalent *GNAS* mutations, has been recognized to represent a quite different biological behavior compared to other histological subtypes^20–22^. A representative feature of intestinal subtype is mucin-hypersection, associated with a dilated papilla with mucin extrusion^23^. Intestinal subtype also tended to be observed in main-duct type with high-grade dysplasia^21^. Furthermore, once IPMN with intestinal subtype invade to stroma, invasive component act as a mucinous carcinoma, better prognosis than tubular carcinoma derived from IPMN with other histological subtypes^24^. On the other hand, pancreatobiliary subtype IPMNs are known to have aggressive biological behaviors with inconspicuous mucin secretion and highly prevalent *KRAS* mutation^21,24,25^, explaining why two IPMN cases with HGD/INV (case #90 and #111) harboring the *KRAS* mutant in cfDNA showed wild-type *GNAS* genotype in the primary lesions. Taken together, as shown in Figs. 3 and 4, we classified the histological subtypes in terms of the mucin-hypersecretion and compared intestinal with non-intestinal subtypes. Notably, the present study supports one possible mechanism that the mucin-hypersecreting IPMNs induce local tissue disruption with acellular mucin pool containing high abundance *GNAS* mutant alleles, then involve the local microvessels, and finally enter the bloodstream. Further investigation is required to explore whether the “mechanical” tissue disruption, characterized by *GNAS* mutation, is more prone to induce bloodstream entry than the “invasive” tissue disruption with *KRAS* mutation.

Based on the strong evidence that the recurrent *GNAS* mutation is specific for IPMN^26,27^, recent studies investigated *GNAS* mutation detection in other biological specimens such as cyst fluid and duodenal and pancreatic juice samples of patients with PCNs^15,28–31^. These studies revealed that a higher positive prevalence of *GNAS* mutation than reported in the present study. In fact, mutation detection sensitivity depends on the anatomical distance from the primary lesion and the lower positive prevalence in peripheral blood samples than in the cyst fluid and juice samples might be inevitable. Nevertheless, blood sample is easier to harvest and is less invasive and can be more applicable to pancreatic screening. Theoretically, combination of blood-based screening with cyst-based detailed examination appeared to be the best diagnostic approach. To overcome low sensitivity, a combination of *GNAS* mutation positivity with novel biomarkers can be explored in future studies.

This study is based on prospectively collected samples harvested from the consecutive patients, leading to minimal selection biases. The most critical limitation of this study is the small sample size, particularly non-IPMN samples such as MCN, SPN, and SCN. The second major limitation of our study is the lack of patients undergoing surveillance. This study include only patient undergoing pancreatic resection and therefore, the use of a surgical case series leads to an enriched study population for IPMN with HGD/INV (27 of 34 cases, 79%). The detection of *GNAS* mutations in cfDNA before the emergence of a visible IPMN highlights the potential of liquid-biopsy to help in the risk stratification of patients undergoing pancreatic screening and surveillance.

In conclusion, we revealed that the detection of *GNAS* mutations in cfDNA from peripheral blood of patients with pancreatic cysts is a highly specific indicator for IPMNs, specifically the intestinal subtype. In the future, the prospective large-scale validation including patients with PCNs being considered for resection or surveillance is needed.

## Materials and methods

### Patients and specimens

From November 2017 to September 2019, 57 consecutive patients with PCNs who underwent surgical resection in Tohoku University Hospital were prospectively enrolled in our observational study and biological specimens such as peripheral blood, cyst fluid, and tissue samples were collected. This study was approved by the Ethical Committee for genetic studies of the Tohoku University Graduate School of Medicine (institutional review board approval number is 2019-1-119). All eligible patients during the course of the study gave written informed consent prior to participation. All research was performed in accordance with relevant guidelines and regulations.

Patients’ blood samples were obtained in the week before surgery using PAX gene ccfDNA tube (PreAnalytiX, Hombrechtikon, Switzerland). The postoperative blood samples were collected just before the discharge. Regarding the resected specimens, we basically used the surgically aspirated cyst fluid samples; however, not all IPMN cases had surgically aspirated cyst fluid samples due to the technical difficulty of sampling or insufficient DNA yields. Only for cases with unavailable cyst fluid samples, we evaluated the mutations using the resected tissue samples. All cyst fluid samples were aspirated from the resected specimens in the operating room immediately after the pancreatic resection using a fine needle sterile syringe and were transferred on ice to the laboratory generally within one hour, where it was aliquoted and stored at − 80 °C until further use. Blood samples were immediately processed to isolate plasma by centrifugation at 1900*g* for 15 min at room temperature and plasma samples were aliquoted and stored. All of cyst fluid, blood, and tissue samples were subjected to linkable anonymization. All experiments were conducted in a blinded fashion, without any prior knowledge of pathological diagnosis.

### Clinical and histological data assessment

Demographic, pathological, and clinical data were collected from a prospectively maintained database and medical records. All resected specimens were histologically evaluated by two of the authors (Y.O. and T.F.) specializing in pancreas pathology and graded as either LGD, HGD or INV based on criteria defined by a recent consensus^32^. Histological subtypes based on the morphological findings of intraductal proliferation and mucin expression were classified as described previously^20^. As a semi-quantitative histological evaluation, we defined the histology score as the number of slide sections with presence of specific findings, such as acellular mucin pool and rupture of pancreatic ducts.

### DNA extraction

Before DNA extraction, second centrifugation for further plasma clearance was performed at 16,000*g* for 10 min at 4 °C to remove cellular debris. The cfDNA was extracted from 4 mL of plasma using the QIAamp Circulating Nucleic Acid Kit (QIAGEN, Hilden, Germany) according to the manufacturer’s instructions. Genomic DNA from cyst fluid (DNeasy blood and tissue kit, QIAGEN) and the formalin-fixed paraffin-embedded tissue (QIAamp DNA FFPE Tissue Kit, QIAGEN) were also extracted. Extracted DNA was quantified by a SYBR Green real-time PCR based method (LINE1-assay) in order to assess the amount of amplifiable DNA as described elsewhere^33^. Standard calibration curve was valid for fivefold serial dilution of human genomic DNA (Promega, Madison, WI).

### Droplet digital PCR

*KRAS* mutation in cfDNA were examined using droplet digital PCR (ddPCR) system and the ddPCR *KRAS* multiscreening kit (Bio-Rad, Hercules, CA), which covers seven types of mutations in *KRAS* codon 12/13 (G12A, G12C, G12D, G12R, G12S, G12V and G13D). Mutation of *GNAS* p.R201C and p.R201H were also examined using respective PrimePCR™ ddPCR™ Mutation Assay (Bio-rad). Because the input cfDNA amount affects the detection sensitivity, we adjusted the loading cfDNA amount with dilution and applied ~ 10 ng into ddPCR as a template. In some cfDNA samples with relatively low quantity, maximum volume of 8.0 μL was applied. Pre-mix preparation, droplet generation, and thermal cycling were performed according to the manufacturer’s instructions. The fluorescence intensity in droplets was detected by a QX200 Droplet Reader (Bio-Rad). For all assays, no template controls were run to determine lack of contamination. We also used the DNA from PANC-1 and BxPC-3 cells as positive controls of *KRAS* mutant and wild, respectively (Supplementary Figure S3). Positive controls of *GNAS* wild type and mutant (R201C and R201H) were obtained as plasmids after the T/A cloning of PCR products from primary resected IPMN tissue samples. QuantaSoft version 1.7.4 analysis software (Bio-Rad) was used for data acquisition and analysis. Only tests providing more than 10,000 droplets were used for analysis. The threshold for distinguishing positive from negative droplets was manually determined. Specific amplification of wild type and each mutant of *GNAS* were validated using multiple positive and negative control samples (Supplementary Figure S3). To ensure the specific amplification and detection, all assays with ≥ 2 mutant droplets were considered positive for the *KRAS* and *GNAS* mutation.

### Next-generation sequencing

The targeted sequencing was performed using Ion AmpliSeq technology (Thermo Fisher Scientific Inc., Waltham, MA, USA). and Ion AmpliSeq Cancer Hotspot Panel v.2 (Thermo Fisher Scientific), which contains 207 primer pairs and targets approximately 2800 hotspot mutations for 50 cancer-related genes. Sequencing libraries were prepared using the 5 ng of DNA and Ion AmpliSeq Library Kit Plus with Ion Xpress™ Library Barcode Adaptors (Thermo Fisher Scientific). Amplified libraries were cleaned up by Agencourt AMPure XP Reagent (Beckman Coulter Life Sciences, Brea, CA, USA). Emulsion PCR was performed on Ion OneTouch™ 2 System, followed by the template-positive Ion Sphere Particles enrichment on the Ion OneTouch™ ES Instrument (Thermo Fisher Scientific) according to the manufacturer's instructions. Sequencing was performed on the Ion Torrent Personal Genome Machine (PGM; Thermo Fisher Scientific) using 316v2 chips. Post-sequencing data processing, including alignment to the hg19 human reference genome and variant calling, were conducted by Torrent Suite version 5.0 software and Torrent Variant Caller (Thermo Fisher Scientific). Alignments and putative mutations were visually verified using the Integrative Genomics Viewer (IGV, v2.3; Broad Institute, Cambridge, MA, USA).

### Statistical analysis

All statistical analysis was performed using the JMP Pro 15.0.0 statistical software (SAS Institute Inc., Cary, NC, USA) and GraphPad Prism Version 8.4.3 (GraphPad Software, San Diego, CA, USA). Continuous and categorical variables were reported as medians and as whole numbers and percentages. We used the non-parametric Mann–Whitney *U* test to compare continuous variables. Categorical variables were analyzed using the Fisher's exact test. *P* values < 0.05 was considered statistically significant.

## Supplementary information

Supplementary Information
