# Supplementary material for: GNAS mutation detection in circulating cell-free DNA is a specific predictor for intraductal papillary mucinous neoplasms of the pancreas, especially for intestinal subtype
Source: Sci Rep. 2020 Oct 20;10:17761. doi: 10.1038/s41598-020-74868-2 (PMC7576136; doi:10.1038/s41598-020-74868-2)
Supplement: Supplementary file 1 — Supplementary Information [file 41598_2020_74868_MOESM1_ESM.pdf]

Supplementary Information for

“*GNAS* mutation detection in circulating cell-free DNA is a specific predictor for intraductal papillary mucinous neoplasms of the pancreas, especially for intestinal subtype”

by

Tatsuo Hata,[1)] Masamichi Mizuma,[1)] Fuyuhiko Motoi,[1)] Yuko Omori,[2)] Masaharu Ishida,[1)] Kei Nakagawa,[1)] Hiroki Hayashi,[1)] Takanori Morikawa,[1)] Takashi Kamei,[1)] Toru Furukawa,[2)] Michiaki Unno,[1)]

1) Department of Surgery, Tohoku University Graduate School of Medicine

2) Department of Investigative Pathology, Tohoku University Graduate School of Medicine

Supplementary Figure S1

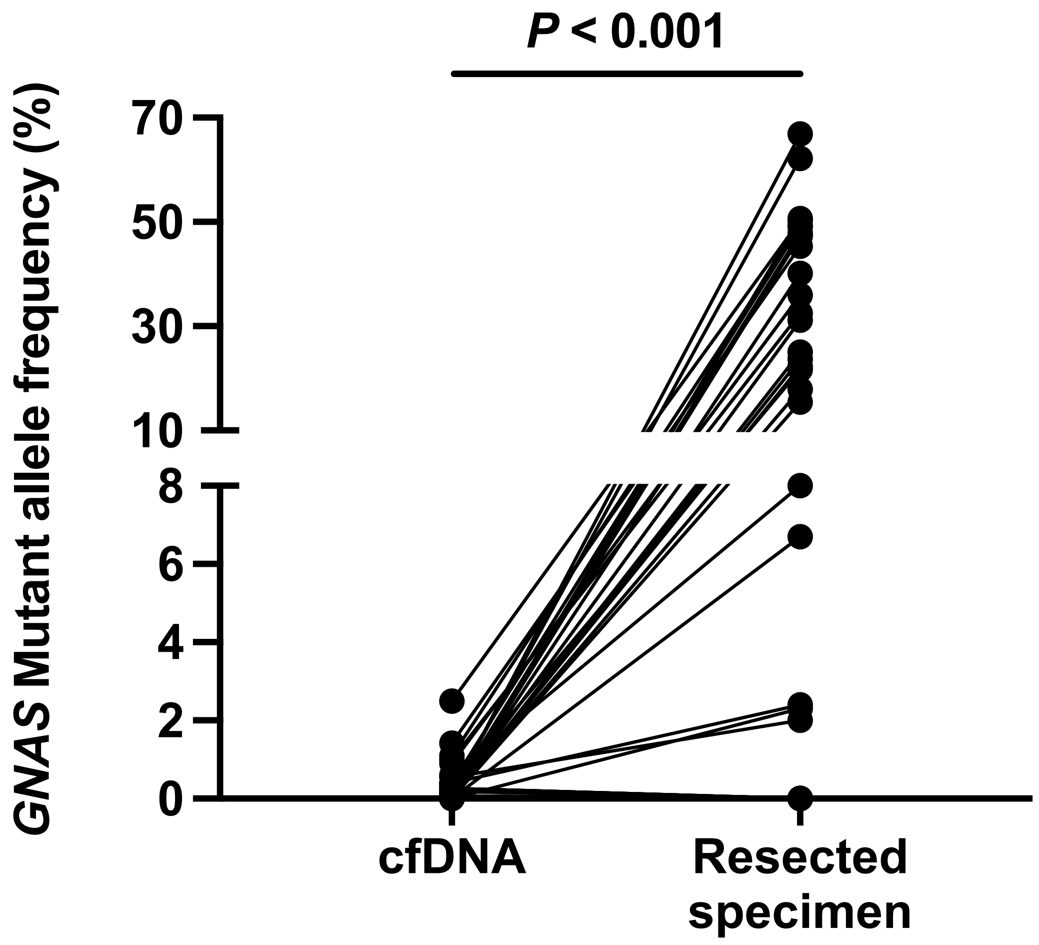

Supplementary Figure S1. Paired comparison of mutant allele frequencies between cfDNA and resected specimen.
cfDNA, circulating cell-free DNA.

Supplementary Figure S2

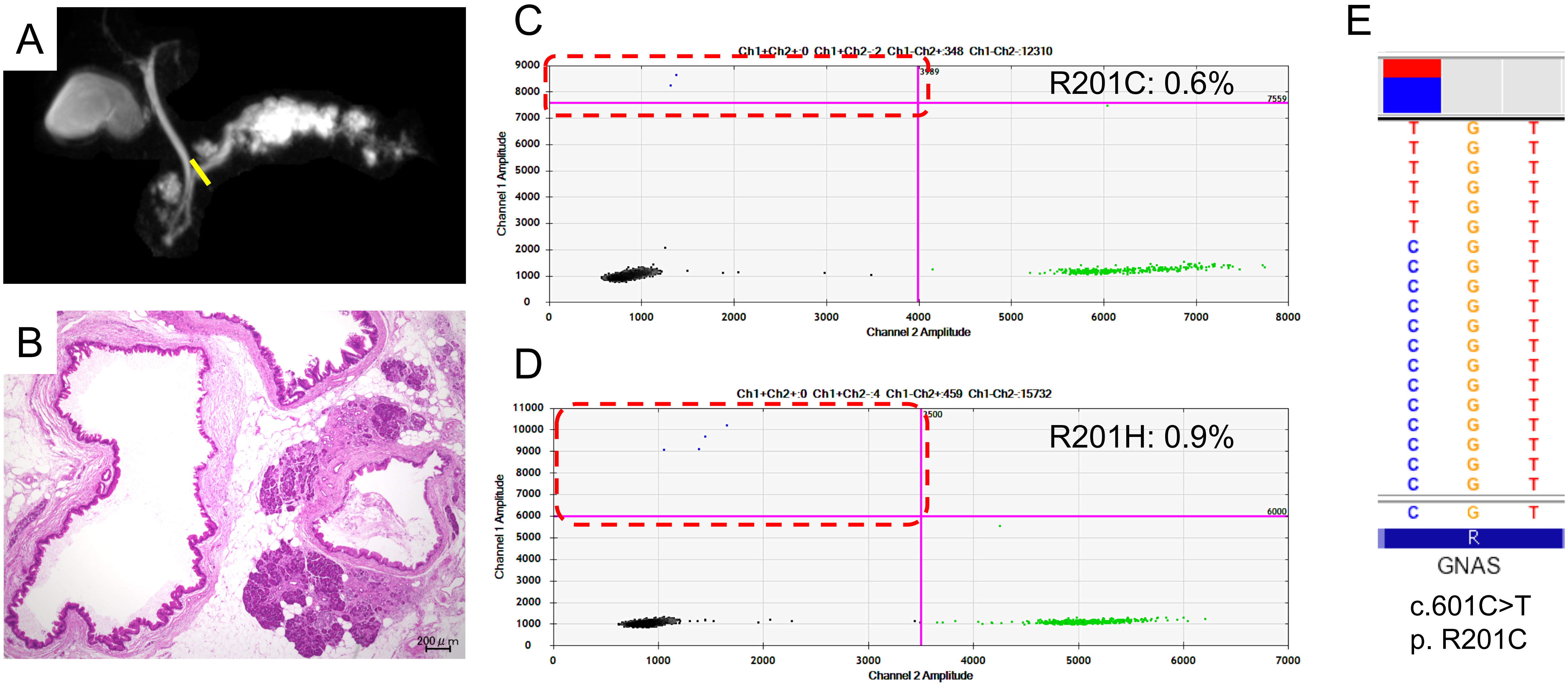

Supplementary Figure S2. A case of multiple branch-duct IPMNs undergoing distal pancreatectomy for the high-risk IPMNs located in pancreas body and tail. (A) Preoperative magnetic resonance pancreatography. Yellow line is the resection line. (B) Histological findings of resected pancreas showing the low-grade dysplasia with gastric subtype. Droplet digital PCR results for *GNAS* R201C (C) and R201H (D) in cfDNA from the preoperative plasma samples. (E) Results of next-generation sequencing analysis using the resected specimen. Primary IPMN lesions harbored only R201C mutational status.

Supplementary Figure S3

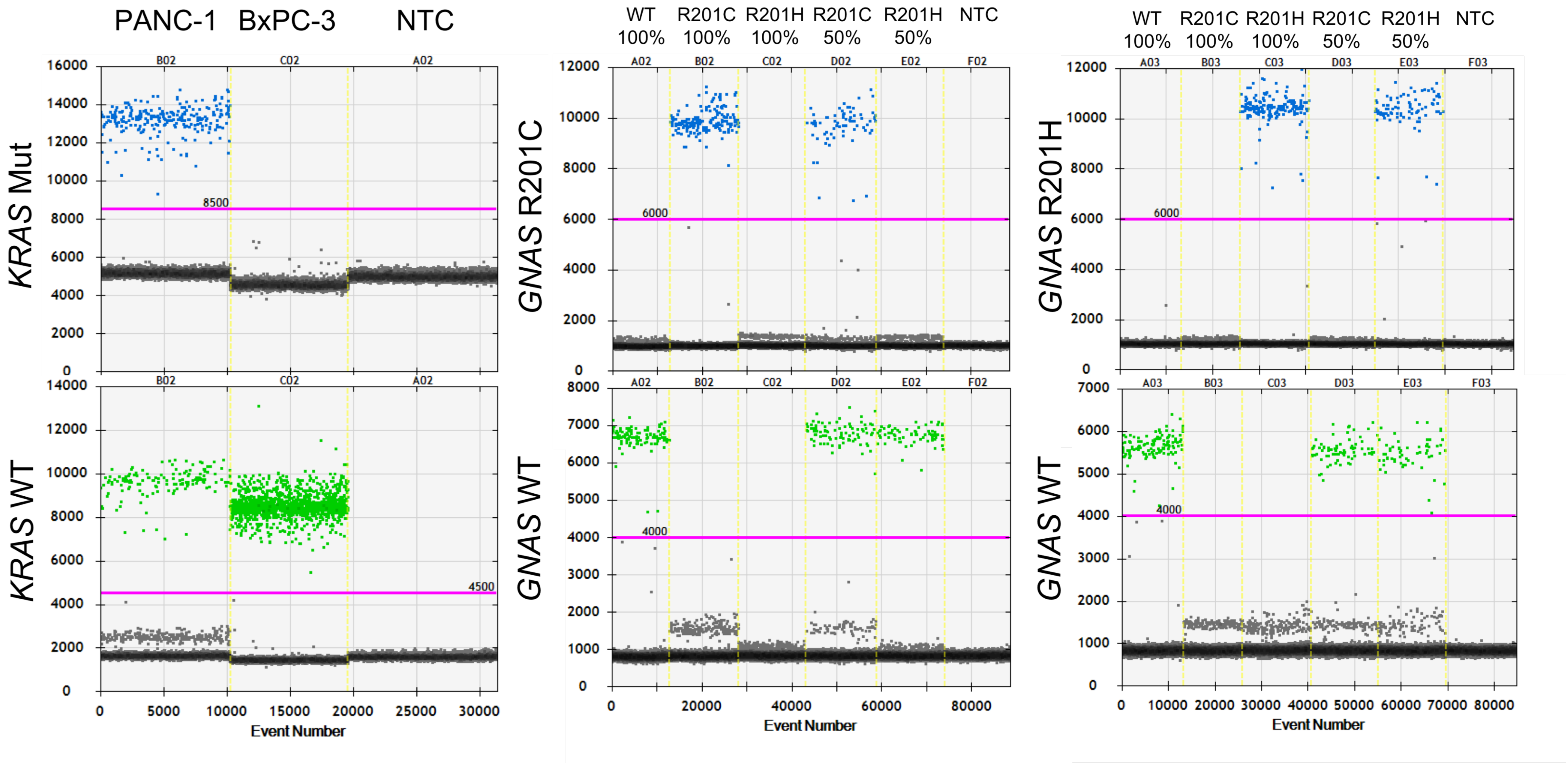

Supplementary Figure S3. Droplet digital PCR results showing the verification of primers/probe targeting *KRAS* and *GNAS* mutation using multiple positive and negative control samples. Pink lines showed the thresholds of fluorescent value of FAM (blue droplets, mutant type) and HEX (green droplets, wild-type).

Supplementary Table S1. Clinical findings of 34 IPMN patients with or without *GNAS* mutation in cfDNA.

Features	cfDNA *GNAS* positive (n = 11)		cfDNA *GNAS* negative (n = 23)		*P*
Sex (n)					0.295
Male	8		12		
Female	3		11		
Age, median (range), year	70	(53–87)	70	(46–83)	0.765
Cyst size, median (range), mm	30	(10–40)	38	(10–90)	0.125
Mural nodule ≥5mm (n)					0.465
Present	8		13		
Absent	3		10		
Dilatation of MPD ≥10mm (n)					0.079
Present	5		3		
Absent	6		20		
Dilatation of MPD ≥5mm (n)					0.999
Present	7		14		
Absent	4		9		
Morphological duct type (n)					0.425
MD predominant	4		5		
BD predominant	7		18		
Serum CEA (ng/mL)	2.9	(0.8–4.4)	2.1	(0.4–9.7)	0.092
Serum CA19-9 (IU/L)	16.7	(0.6–52.0)	10.9	(0.6–164.1)	0.330

cfDNA, circulating cell-free DNA; MPD, main pancreatic duct: MD, main duct; BD, branch-duct.
